# A case series of benign transient hyperphosphatasemia from a pediatric endocrinology reference health facility in Turkey

**DOI:** 10.11604/pamj.2018.30.206.15754

**Published:** 2018-07-11

**Authors:** Fatma Dursun, Heves Kirmizibekmez

**Affiliations:** 1 Department of Pediatric Endocrinology, Umraniye Training and Research Hospital, Istanbul, Turkey

**Keywords:** Benign, transient, hyperphosphatasaemia, alkaline phosphatase

## Abstract

**Introduction:**

Benign transient hyperphosphatasemia (BTH), even a known condition, is not very well managed by primary care physicians. The diagnostic criteria for BTH were alkaline phosphatase (ALP) levels above 3-5 times greater than the age adjusted upper limit of normal among children under 5 years with no evidence of liver or bone disease whose ALP values resolved within 4 months.

**Methods:**

This study involved 15 patients aged 0-5 years, who were referred to the pediatric endocrinology clinic for elevated ALP levels. They were diagnosed with BTH. We examined demographic and biochemical parameters including ALP and ALP isoenzymes, liver enzymes, calcium, phosphate, and parathormone (PTH) levels to rule out liver or bone disease as a cause for hyperphosphatasaemia.

**Results:**

Of 15 patients 7 were male and 8 were female. Mean age was 2.45 ± 1.09 (range 1.2-4.6) years. Mean serum ALP level was 2315 ± 1028 IU/L (1102-4662), while liver enzymes, calcium, phosphate, PTH and vitamin D3 levels were in normal ranges. The mean normalization period of ALP was 2.4 ± 1.1 (0.5-4) months, and all were normal at the end of 4 months without any treatment.

**Conclusion:**

This study and literature knowledge related to BTH has shown that being aware of BTH is very important for a primary care physician. Paediatricians can conveniently manage the differential diagnosis and follow up this period of elevated ALP.

## Introduction

Alkaline phosphatase (ALP) is produced in liver, bone and placenta, and is normally present in high concentrations in growing bone and in bile. Serum ALP is measured to diagnose or monitor diseases in the skeleton or hepatobiliary system. In healthy adults, the major activity of ALP is represented by liver and bone isoforms, while in healthy infants and children, as a result of growth, the serum is rich in the bone isoform of ALP [1]. The serum level of ALP changes with age. It is mildly higher than adult levels during the first 3 months of life, increases at puberty two-to threefold and remains above the adult level for one to two years. This increase is related to the bone growth spurt during puberty [2]. Given the possibility of underlying asymptomatic hepatobiliary or bone diseases, these patients are usually referred to either a paediatric gastroenterologist or a paediatric endocrinologist for evaluation and management. Importantly, in a significant proportion of these patients, elevated ALP is benign, and gradually resolves without any treatment, questioning the rationale for an exhaustive evaluation of every patient with this biochemical abnormality. The benign elevation of ALP is referred to as benign transient hyperphosphatasaemia (BTH), a condition most commonly observed in infants and children younger than 5 years of age [3]. Characteristic features of BTH defined by Kraut [4] et al including; age of presentation less than five years; no other evidence for bone or liver disease on physical examination or laboratory findings; elevation in both bone and liver ALP isoenzymes; and a return to normal serum ALP values within four months. Incorporating Kraut's original diagnostic criteria, updated with more recent relevant literature, a presumptive diagnosis of BTH was made by Chu [5] et al with the following: significantly elevated ALP level (median: 9 times the upper limit of normal); age < 5 years; history and physical examination not suggestive of bone or liver disease; normal liver tests; normal electrolytes, calcium, blood urea nitrogen and creatinine; ALP isoenzymes show an absolute elevation of both bone and liver fractions, but the relative predominance may be of bone, liver, or mixed origin; normal PTH and 25-hydroxy vitamin D levels; confirm BTH with a normalizing ALP level within 3 to 4 months. In this study we described clinical features of patients who were referred to paediatric endocrinology for elevated ALP and discussed in the light of literature related to BHT. The aim was to give information about the relationship between clinical features and recovery time.

## Methods

This retrospective study involved 15 patients aged 0-5 years who were brought to the pediatric endocrinology outpatient clinic between January 2014 and December 2017 because of elevated ALP levels and diagnosed with BTH. Ethical approval was obtained from the hospital ethics committee (approved number: 2925-date: 17.01.2018) and written informed consent was obtained from parents or legal guardians of each patient. We examined demographic variables as well as biochemical parameters including ALP and ALP isoenzyme levels, liver enzymes, calcium, phosphate, 25-hydroxy vitamin D3 and PTH levels to rule out liver or bone disease as causes of hyperphosphatasaemia. The diagnostic criteria for BTH were ALP levels above 3-5 times greater than the age adjusted upper limit of normal among children <5 years with no evidence of liver or bone disease whose ALP values resolved within 4 months. Upper limit of ALP given by our central biochemistry laboratory was 350 IU/L for both girls and boys between 1 and 5 years. Laboratory tests also included the following; alanin aminotransferase (ALT), aspartate aminotransferase (AST), gamma-glutamyl transpeptidase (GGT), calcium, phosphate, magnesium, blood urea nitrogen, creatinine, ALP, 25-hydroxy vitamin D3, PTH, isoenzymes of ALP. ALT and AST levels were studied using Abbott kits and an Abbott i8000 model device at our hospital's central laboratory. ALT and AST were measured with the enzymatic method. PTH and 25-hydroxy vitamin D3 levels were measured with an Abbott i16000 model device using the “Chemiluminescent microparticle immunoassay (CMIA)“ method. ALP was measured with Abbott Aeroset/C4000/C8000/C16000 system device using the colorimetric assay with a standardized method using p-nitrophenyl phosphate as substrate and 2-amino 2-methylpropanol as buffer. ALP isoenzymes were measured with gel electrophoresis method.

**Statistics**: Study data were analyzed using IBM SPSS Statistics 22 (IBM SPSS, Turkey) software package. Descriptive statistics included mean and standard deviation.

## Results

Fifteen patients (7 males and 8 females) referred to our clinic between 2014 and 2017 with elevated serum ALP levels were included in this study. The diagnosis of BTH was made in each patient based on their clinical and biochemical features (Table 1). Mean age was 2.45 ± 1.09 (1.2-4.6) years. There was not any patient under one year old. The mean weight SDS was 0.51 ± 1.1, and the mean height SDS was 0.54 ± 1.1. Only one patient had a history of acute gastroenteritis one week prior to referral, while others did not have any evidence of additional illness. One patient was under the treatment of L-thyroxine for congenital hypothyroidism; however he was euthyroid at referral. In addition, two patients had failure to thrive and 4 patients had the complaint of leg pain (Table 1). None of them had a physical examination finding suggesting rickets, other metabolic bone disease, liver or biliary system disease. Serum ALP levels were between 1102 and 4662 IU/L (2315 ± 1028), and calcium, phosphate, urea nitrogen, creatinine, PTH, AST, ALT, GGT, 25-OHD3 levels were all in normal ranges (Table 2). A total of 11 patients had documented elevated “bone-specific isoenzyme of ALP“ levels. Bone-specific isoenzyme of ALP levels were reported as >90 IU/mL, confirming that especially bone-isoenzyme generates this elevated total ALP levels in these patients. Normalization period for ALP was between 2 weeks and 4 months (mean 2.4 ± 1.1 month) in our patients.

**Table 1:** Clinical and laboratory findings of 15 patients with benign transient hyperphsophatasemia

PN	Age at diagnosis (year)	Sex	Weight SD	Height SD	Clinical features	Ca mg/dl	P mg/dl (N:4.5-7.5)	ALP IU/L (N<350)	PTH pg/ml (N:11-65)	25OHD3 ng/ml (N>12)	ALP-bone IU/ml (N<90)	NP month
1	1.2	F	0.2	0.2	Inability to walk	10.3	5.3	2290	20.9	40	>90	3
2	2.7	F	2.6	0.8	Normal	9.9	4.9	3272	28	21.5	>90	3
3	4.6	F	-2.4	-0.4	Failure to thrive	10.7	5.1	1933	33	19.3	>90	3
4	2.1	F	1.02	0.1	Leg pain	10.6	6.2	1645	44	19	NA	1.5
5	2.1	F	1.89	2.2	Normal	10.5	6.3	4662	60	13	>90	0.5
6	1.6	M	1.5	1.6	Normal	10.1	4.8	1807	12	26.8	NA	1
7	2.4	M	0.17	1.2	Normal	9.7	5.3	2485	22	24.7	>90	2
8	3	M	0.1	-1.5	Normal	9.9	4.8	1932	24	41	>90	2
9	3.3	M	0.2	0.3	Leg pain	9.5	4.8	3904	38	23.1	>90	4
10	1.3	M	-0.17	-1.6	Failure to thrive	9.6	5.7	1130	60	23.6	>90	4
11	1.8	M	-0.21	1.5	Delay in closure fontanell	9.8	5.1	3300	18	32.7	>90	1
12	1.6	F	0.14	-0.2	Normal	10.2	5.8	1817	49	17.7	NA	2
13	1.2	M	1.19	1.3	Normal	9.2	5.3	1600	61	15.5	>90	3
14	4	F	0.12	1.1	Normal	9.6	5.5	1102	11	16.8	NA	4
15	3.9	F	1.3	1.5	Leg pain	9.9	5.3	1850	13	25.9	>90	4
												

PN: patient number, F: female, M: male, SD: standart deviation , Ca: calcium, P: phosphate, ALP-bone: alkaline phosphatase bone isoenzyme, NA: Not available, NP: normalization period

**Table 2:** Anthropometric and laboratory data of children with benign transient hyperphosphatasemia

	Subjects (Mean±SD)
Age (years)	2.45±1.09
Female, n (%)	8 (53. 3%)
Male, n (%)	7(46. 7%)
Weight (SD)	0.51±1. 1
Height (SD)	0.54±1. 1
Calcium (mg/dL)	9.9±0. 4
Phosphate (mg/dL)	5.3±0. 4
25OHD3 (ng/mL)	24±8. 3
PTH (pg/mL)	32.8±18
ALT (IU/L)	19.6±8. 2
AST (IU/L)	28.4±8. 3
GGT (IU/L)	17.3±9. 3
ALP (IU/L)	2315±1028
ALP- isoenzyme	>90
Normalization period (month)	2.4±1.1

SD: standardize deviation , PTH: parathormone, ALT:alanin aminotransferase,

AST: aspartate aminotransferase , GGT: gamma-glutamyl transpeptidase, ALP: alkaline phosphatase

## Discussion

In this report, we describe 15 otherwise healthy children with BTH with no evidence of liver or bone disease. ALP is a commonly employed biochemical marker offered by clinicians to screen for skeletal or hepatic disorders. Therefore high ALP levels are potentially a common cause of referral to tertiary care centers for further evaluation. In this study we underlined the importance of correct evaluation of ALP levels, which varies due to age, physiologic events such as rapid growth and puberty. The importance of considering BHT, a benign and transient condition of childhood, was also highlighted. The primary diagnostic concerns when encountering elevated ALP levels in children include; bone disorders, liver disease and less commonly kidney disease and drug reactions (anticonvulsants, antibiotics) [1,5]. In the primary care setting, the emphasis should be on clinical assessment supplemented by limited laboratory investigations [3]. In case of elevated ALP levels, the history should focus on symptoms of liver, intestinal, renal, and bone diseases with careful evaluations of any drug administrations-prescribed as well as over the counter. Specific inquiries should be made about the use of anticonvulsants such as phenobarbital, phenytoin or carbamazepine [6]. Symptoms indicating hepatobiliary disease such as dark colored urine, pruritus, and steatorrhea should be carefully evaluated. Similarly bossing, widening of the wrist should be carefully looked for [3]. BTH is considered a rare, non-recurring condition that affects healthy infants and children of both sex and resolves within 4 months without intervention [7, 8]. Due to the typical age, normal physical examination, and elevated of both liver and bone-specific ALP-isoenzymes the diagnosis of BTH was made according to the commonly used criteria [4]. The age criterion proposed by Kraut [4] et al suggests that the diagnosis of BTH should be considered in children below 5 years of age. In a recent review, the median age of presentation of BTH was 18 months with no gender predominance [7]. In another study Teitelbaum [9] et al reported that the average age of their patients was 2.5 years. In our study, the median age of presentation of BTH was 2.45 ± 1.09 years with no gender predominance, consistent with previous reports. None of our patients were under one year old, which was the only difference of our series. The peak value of ALP in BTH is usually very high (3-30 fold above upper reference ranges) in comparison with other causes of ALP elevation [1]. In a recent study, the ALP was elevated at least 5 times normal in 71% of the patients with BTH [7]. Intermediate elevations of less than 5 times the normal may possibly represent either developing or resolving BTH [10]. In our study group, ALP levels were at least three times the normal upper limit (between 1102 and 4662 IU/L).

In a study estimating the frequency of BTH, ALP was measured at 3 time points: ages 2 to 4, 4 to 7, and 1 to 15 months. The number of new cases detected at each time point were 3 of 260 (1.2%) at 2 to 4 months of age, 2 of 186 (1.1%) at 4 to 7 months of age, and 3 of 85 (3.5%) at 11 to 15 months of age [10]. In a cohort of 321 healthy infants and toddlers' age 8-24 months investigated in Boston, 2.8% were found to have a transient and unexplained elevation of the ALP level [11]. BTH is often discovered incidentally with a routine blood analysis or with various illnesses when laboratory studies are obtained for another purpose [5]. BTH is a benign disorder believed to be triggered by an infectious disease, most probably viral infections [12]. Common viral infections, such as respiratory infections or gastroenteritis as in one of our patients and specific infection such as enterovirus, and Epstein-Barr virus have all been reported [7, 13, 14]. Other associated conditions reported in the literature have included failure to thrive, HIV, patients with a liver or kidney transplant [15-17]. However, diagnosing BTH in these children is certainly more difficult. A causal relationship with any of these and BTH has not been definitively established. We did not systematically test for the presence of an infectious disease; however patients did not have any physical sign or symptom of an infection. According to past medical history, only one patient had a history of acute gastroenteritis, two patients had failure to thrive and 4 patients had the complaint of leg pain. The pathogenesis of BTH remains poorly understood. There is no evidence of vitamin D deficiency, increased bone turnover or resorption as the cause. Electrolytes and hormones related with bone metabolism are usually normal. Increased production of ALP in its tissues of origin (most probably in bone), increased activation of circulating ALP and impaired clearance of ALP from the circulation have all been proposed as the mechanism responsible for BTH [1, 5]. Calcium, phosphorus, 25-hydroxy vitamin D3 and PTH levels of our patients were all in normal ranges. Bone specific ALP isoenzyme levels of eleven patients in our group were elevated. In a recent study, Gualco [7] et al showed that, ALP levels return to normal in a median of 10 weeks, with 80% of patients having normal ALP within 4 months. Given the benign nature of this entity, rechecking ALP earlier than four months is defined as unnecessary [4]. Normalization time for ALP was also not longer than 4 months in our study group, regardless of how high the ALP levels were. As the result, we could not be able to add a new comment to the diagnostic criteria of BHT. Also, clinical progress of patients in this group showed an uneventful recovery in the expected time consistent with previously reported cases.

## Conclusion

ALP is among the most common laboratory tests that general practitioners order to evaluate the presence of rickets, bone or liver disorders in clinical practice. This study showed the importance of awareness and clinical course of BTH. Paediatricians can conveniently manage the differential diagnosis and follow up this period of elevated ALP. The evaluation of the patient with a detailed history, an accurate physical examination and baseline laboratory tests is generally sufficient to exclude possible liver or bone disease. ALP level normalizes in four months no matter how high it is. We think that this is a good example of a basic medicine lesson; “laboratory tests should not come before physical examination and clinical evaluation“.

### What is known about this topic

- Benign transient hyperphosphatasemia (BTH), previously reported as case reports, is a benign-transient condition of childhood. Even reported in the literature and takes a small place in textbooks; it is not very well-known and managed in daily clinical practise.

### What this study adds

- The marked elevation in ALP levels may be worrying for physicians and parents; BTH is rare and unfortunately present with an “alarmingly high“ laboratory finding, causing anxiety, unnecessary repeated investigations and superior center referrals;
- In this study we described clinical features of patients who were referred to paediatric endocrinology for elevated ALP and discussed in the light of literature;
- We concluded that “no matter how high, ALP becomes normal in four months“; the aim was to make guidance for young physicians for effective management of BTH.

## Competing interests

The authors declare no competing interest.
